# Unifying concepts in methanogenic, aerobic, and anammox sludge granulation

**DOI:** 10.1016/j.ese.2023.100310

**Published:** 2023-08-10

**Authors:** Simon Mills, Anna Christine Trego, Marco Prevedello, Jo De Vrieze, Vincent O’Flaherty, Piet N.L. Lens, Gavin Collins

**Affiliations:** a Microbial Communities Laboratory, School of Biological and Chemical Sciences, National University of Ireland Galway, University Road, Galway, H91 TK33, Ireland; b Microbial Ecology Laboratory School of Biological and Chemical Sciences, University of Galway, University Road, Galway, H91 TK33, Ireland; c Center for Microbial Ecology and Technology (CMET), Ghent University, Coupure Links 653, B-9000, Gent, Belgium; d University of Galway, University Road, Galway, H91 TK33, Ireland

**Keywords:** Granule, Granulation, Anaerobic digestion, Anammox, Aerobic granule

## Abstract

The retention of dense and well-functioning microbial biomass is crucial for effective pollutant removal in several biological wastewater treatment technologies. High solids retention is often achieved through aggregation of microbial communities into dense, spherical aggregates known as granules, which were initially discovered in the 1980s. These granules have since been widely applied in upflow anaerobic digesters for waste-to-energy conversions. Furthermore, granular biomass has been applied in aerobic wastewater treatment and anaerobic ammonium oxidation (anammox) technologies. The mechanisms underpinning the formation of methanogenic, aerobic, and anammox granules are the subject of ongoing research. Although each granule type has been extensively studied in isolation, there has been a lack of comparative studies among these granulation processes. It is likely that there are some unifying concepts that are shared by all three sludge types. Identifying these unifying concepts could allow a unified theory of granulation to be formed. Here, we review the granulation mechanisms of methanogenic, aerobic, and anammox granular sludge, highlighting several common concepts, such as the role of extracellular polymeric substances, cations, and operational parameters like upflow velocity and shear force. We have then identified some unique features of each granule type, such as different internal structures, microbial compositions, and quorum sensing systems. Finally, we propose that future research should prioritize aspects of microbial ecology, such as community assembly or interspecies interactions in individual granules during their formation and growth.

## Highlights

• EPS, especially protein content, is important for granulation in all three sludge types.
• Physio-chemical influences on granulation are shared across all three sludge types.
• Biological aspects of granulation differ between granule types.
• Anammox granulation occurs via the aggregation of microgranules.
• Methanogenic and aerobic granules undergo concentric growth.

## Graphical abstract

**Figure:**
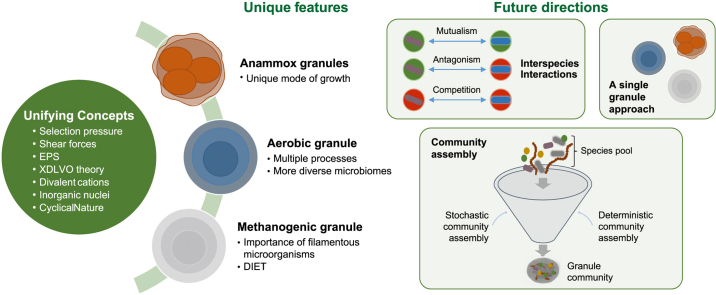

## 1 Introduction

Biological wastewater treatment relies on microbial biomass for carbon degradation and the transformation of macronutrients, such as nitrogen and phosphorus. The cultivation and retention of active biomass engaged in decomposition and nutrient cycling in treatment systems is essential. The most widely applied and successful biological treatment method is the activated sludge process, which relies upon the breakdown of organic contaminants by heterotrophic microorganisms [1]. Activated sludge is flocculent in nature, and its cultivation requires active aeration, which leads to some drawbacks, namely, large physical footprints and high capital and operational costs [2].

Granular sludge-based bioreactors provide an alternative to the activated sludge process. In such bioreactors, microbial biomass grows in the form of granules, which are small, self-immobilized, spherical, microbial aggregates of 0.5–4 mm in size [3]. Granules are denser and more compact than activated sludge flocs, and granular sludge bioreactors require less surface area and lower up-front capital costs [2]. Settling velocities for granules are typically faster than activated sludge flocs [3]. This permits the uncoupling of hydraulic retention and biomass retention times in granule-based systems, allowing for high-rate wastewater treatment.

Granular biomass is most commonly applied in three forms: methanogenic granules, aerobic granules, and anammox granules. Other more recent applications include photogranules [4], hydrogenic granules [5], algal/bacterial granules [6], and fermentative granules [7]. However, the application of these granule types is still in the experimental phase, and the volume of literature specific to granulation is relatively small compared with methanogenic, aerobic, and anammox granules.

Methanogenic granules were initially applied for anaerobic digestion (AD) in upflow anaerobic bioreactors, such as the upflow anaerobic sludge blanket (UASB) [8] in the 1980s. These bioreactors use the naturally occurring, microbially-mediated AD pathway to convert organic contaminants in wastewaters to valuable biogas, and are now widely applied for the treatment of a variety of industrial wastewaters [9].

Aerobic granules were developed more recently [10], and are used for heterotrophic removal of carbon from wastewater streams, coupled to nitrogen and/or phosphorus removal. Aerobic granules are typically applied in sequencing batch bioreactors (SBRs) rather than the continuous systems applied for AD. SBRs have traditionally been favoured for aerobic granulation as continuous flow reactors were thought to lead to granule instability [11,12]. This instability has been attributed to a lack of feast-famine cycles or the absence of settling velocity-based selection pressure [13]. However, the continuous operation of aerobic granular sludge systems is an area of ongoing research, and a comprehensive review by Kent et al. [14] provided valuable insights into this topic.

Anammox granular sludge is typically applied for ammonium removal from wastewater. The anammox process involves the autotrophic conversion of ammonium to N_2_ gas with nitrite as a terminal electron acceptor and is mediated by several genera of anammox bacteria [15]. The process is more energy efficient than approaches using conventional denitrification since less aeration is required, and there is no requirement for the addition of external sources of carbon [16]. However, anammox bacteria are very slow growing, with doubling times of up to two weeks [17], meaning that effective biomass retention is essential for stable ammonium removal, which can be achieved through the development of dense, stable anammox granules [18].

The mechanisms by which different granule types form (granulation) have been extensively reviewed [15,[19], [20], [21], [22], [23]]. However, these reviews often focus on individual granule types, overlooking the opportunity to consider granulation mechanisms as a broader concept that transcends specific processes or technologies. Therefore, to the best of our knowledge, there is no universally accepted mechanism or theory that explains granulation across all granule types. Consequently, the overall objective of this article is to review the existing literature around granulation theories of methanogenic, aerobic and anammox granules and to identify commonalities which may contribute to a universal granulation hypothesis. We have also identified different features of each granule type which may make this difficult. In addition, we aimed to propose a new ecological approach to studying granulation, which could be applied to all granule types to find additional commonalities. We hypothesize that granulation theories based on physio-chemical properties or bioreactor operational parameters will share common elements across all granule types, while microbiology-based theories may exhibit slight variations depending on the specific granule type.

## 2 Unifying concepts in granulation

In this section, we present a comprehensive review of several concepts that consistently arise in methanogenic, aerobic, and anammox granulation studies. These concepts have been categorised into three main groups: physio-chemical, operational, and microbial granulation theories.

### 2.1 Physio-chemical granulation theories

#### 2.1.1 Importance of extracellular polymeric substances (EPS)

Several studies state the importance of EPS for the formation of an initial matrix that facilitates granulation and provides structural stability in methanogenic [24], aerobic [25], and anammox [26] granules. A comprehensive, three-step EPS-based theory on anammox granulation was proposed by Lin and Wang [27]. The first step involved initial cell-to-cell contact with anammox bacteria, followed by aggregation in a thin EPS matrix. Next, separate cell clusters aggregate together with other heterotrophic bacteria, which consume the EPS secreted by anammox bacteria and fill in gaps amongst them, helping to form a spherical aggregate. Xu et al. [28] proposed an EPS-based granulation mechanism for methanogenic granules, which relied on a continually decreasing organic loading rate (OLR) and increasing upflow velocity (V_up_) to stress the microbial community and stimulate EPS production, particularly proteins, which improved granule formation. Similar observations regarding increased EPS protein content and granulation were seen in an internal circulation (IC) bioreactor treating leachate from a municipal waste incineration plant [24] and chitosan-supplemented bioreactors treating organic solvent-containing wastewaters [29]. Cui et al. [30] proposed an aerobic granulation mechanism in which heterotrophs grew quickly and established an EPS matrix as the basis for granule formation. Further aggregation of nitrifying organisms into the EPS matrix was then promoted by hydrophobic interactions between their cell surfaces and EPS [18].

High protein-to-polysaccharide ratios in EPS are generally thought to increase hydrophobicity and facilitate better aggregation [31,32]. This link between hydrophobicity, protein content, and improved granulation can be found in all granule types [13,28,33], and therefore, EPS, which is extracted from granular biomass, tends to have hydrophobic properties. The exact mechanism of these hydrophobic interactions is not well established. However, hydrophobic functional groups of some amino acids in the protein fraction of anammox EPS have been suggested to enhance granulation [34]. In addition, a pure culture study of *Pseudomonas stutzeri*, a self-aggregating organism, indicated that the hydrophobic R groups of some amino acids, including tyrosine, leucine, and phenylalanine, promoted aggregation [35].

Two points are common among all EPS-based granulation theories: (1) EPS production forms an initial matrix for the attachment of other cells, and (2) higher protein content is beneficial for granulation. Manipulation of operational parameters has been shown to stimulate EPS production, such as starvation or feast-famine feeding [13], and the application of hydraulic disturbances, including increased upflow velocity [28] or increased duration of aeration time and intensity [36]. Novel methods, such as the application of magnetic fields, have also been shown to promote EPS production in aerobic granules [37]. Specifically, magnetic fields have been observed to increase protein concentrations in EPS [38], which is thought to be caused by iron accumulation within the granule, preferentially binding EPS-protein [39]. However, overproduction of EPS in response to stresses can also harm bioreactor performance by reducing granule settling velocity [40], leading to biomass washout.

#### 2.1.2 Role of cations

EPS in all three sludge types has been found to be negatively charged [[41], [42], [43]]. This is thought to be due to the presence of functional groups, including carboxyls and hydroxyls [44]. The presence of divalent cations, such as Fe^2+^, Ca^2+^, and Mg^2+^, can positively affect granulation by counteracting these negative charges leading to divalent cation bridging with anionic EPS components [20,44,45].

The addition of divalent cations and other cationic polymers has been shown to improve methanogenic granulation and overall bioreactor operation [29,46]. Beneficial cation concentrations are variable, with wide ranges of effectiveness often reported. Fe^2+^ concentrations between 300 and 450 mg L^−1^ have enhanced granulation in UASB reactors [47], whereas concentrations from 100 to 1000 mg L^−1^ have been reported for Ca^2+^ [[16], [48], [49]].

Divalent cationic bridging has been observed as an important aspect of EPS bridging in anammox granular sludge, resulting in dense, stable granules [50]. The addition of Fe^2+^ and Fe^3+^ at concentrations of ≤1.5 mg L^−1^ and ≤ 1.9 mg L^−1^ to stirred bioreactors seeded with non-granular anammox sludge was shown to promote granulation and enhance anammox activity [51]. Wang et al. [52] also found that Fe^3+^ enhanced granulation at concentrations ranging from 2.24 to 7.84 mg L^−1^. The concentration of Ca^2+^ reported for improved anammox granulation is often much higher than the Fe^2+^ concentration. For example, de Graaff et al. [53] reported much-improved granulation when Ca^2+^ concentrations were increased from 42 to 81 mg L^−1^, and Xing et al. [54] found that 152 mg L^−1^ Ca^2+^ improved granulation under salinity stress.

Multiple studies have also implicated the importance of cations, such as Ca^2+^ or Mg^2+^, in aerobic granulation due to their interaction with EPS at concentrations ranging from 10 to 50 mg L^−1^ [[55], [56], [57], [58]]. In contrast, iron concentrations required for improved aerobic granulation were demonstrated to be lower at 1–10 mg L^−1^ [59]. Addition of Ca^2+^ or Mg^2+^ at concentrations of 63 and 21 mg L^−1^, respectively, has even been shown to enhance granulation in halophilic activated sludge, treating saline wastewater, which is a notoriously difficult type of system in which to achieve good granulation [60]. Since saline wastewater is known to cause increased EPS production [61], this could explain the effectiveness of divalent cation addition in achieving granulation in such systems [60]. To summarise, the role of divalent cations in linking negative charges on bacterial surfaces or in EPS appears to be common among all granule types, and the addition of exogenous cations has been shown as an effective strategy for improving granulation. Hence, any universal granulation theory should include the role of cations in the early stages of granulation.

#### 2.1.3 Formation around a nucleus

Inert particulate matter present in influent wastewater has been proposed to act as a nucleus for the initiation of methanogenic sludge granulation by providing a substratum for initial microbial attachment, followed by concentric growth and granule maturation [62,63]. The availability of nuclei in the feedstock, such as inorganic precipitates, has even been suggested to influence the size distribution of granules in bioreactors [63,64].

Inorganic calcium and phosphate precipitates were observed at the core of mature aerobic granules, surrounded by microbial cells attached to an EPS matrix [65]. These precipitates were proposed to act as nuclei for the initiation of granulation under alkaline conditions, followed by microbial growth and concentric growth leading to mature granules [65]. A calcium carbonate core was also observed in aerobic granules by Ren et al. [66], who suggested it provided increased rigidity and structural stability but resulted in lower microbial activity. Other studies, however, have found that organic particulate matter can have no effect [64] or even a negative influence [67] on granulation rates, despite evidence that organic particles become incorporated into aerobic granules before their degradation [68].

Fernandez et al. [69] investigated the effectiveness of inorganic salt precipitation on biomass retention in an anammox SBR and found that increasing NaCl concentrations in the influent led to the precipitation of inorganic salts and improved biomass retention. Retained biomass had a higher inorganic content and increased density, indicating that salt precipitates were acting as precursors for granule formation [69].

The addition of various carrier materials to bioreactors has also been used to promote biomass growth. For example, zeolite addition was effective in improving granulation and biomass retention in methanogenic bioreactors [70]. Granular activated carbon (GAC) has been successfully used as a nucleating agent to accelerate the formation of aerobic granules [71]. Amendment of GAC as a support material for anammox granulation has also successfully shortened start-up times from 109 to 94 d [72]. Fungal spores or mycelial pellets have been demonstrated to improve aerobic granulation by providing a structure on which bacteria can attach and initiate granule formation [73]. The addition of fungal pellets to an SBR sped up granulation by over 30 days compared to non-amended controls [74]. Iron precipitates were formed by applying magnetic fields in a sequencing batch reactor, which acted as a core, and improved granulation in a nitrifying SBR [37].

The addition of carrier materials alters the initial steps of granule formation because this circumvents the need for initial cell-to-cell contact. In fact, when carrier materials are added, granule formation may proceed more like microbial biofilm growth on inert surfaces. The addition of electrically conductive materials, such as GAC, may also promote direct interspecies electron transport (DIET) in methanogenic granules (as discussed in Section 3.2). While inorganic nuclei can help to initiate granulation, and the addition of support material has improved biomass retention, the addition of growth nuclei does not appear to be a prerequisite for granulation.

#### 2.1.4 Granule life cycles

**Fig. 1:**
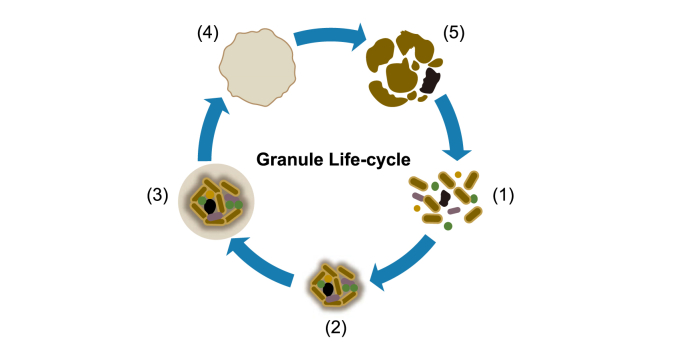
Granule Life-cycle. Depiction of a typical granule life-cycle: (1) broken granule pieces provide the nuclei for new growth; (2) cells aggregate around broken granule pieces in a matrix of EPS and/or filamentous microorganisms; (3) the initial granule/cell cluster grows larger, and it's three-dimensional structure is shaped by reactor hydrodynamics; (4) granules reach a maximum size, and granule stability decreases; and (5) large granules disintegrate, providing nuclei for new granule formation.

Granulation occurs not only in isolation at the outset of bioreactor operation but continually in a dynamic process throughout bioreactor operation [75]. For example, the disintegration of mature granules may also contribute to granulation. Several ‘life-cycle’ theories have been suggested (Fig. 1), in which small granules are considered young and large granules are older [75,76]. Zheng et al. [77] proposed a methanogenic granule life cycle where growth was initiated by clusters of *Methanosaeta concilli.* This was followed by a “coat” of syntrophic bacteria and subsequent concentric growth, resulting in mature granules. Substrate limitation at the centre of large granules leads to decay and breakage, and the resultant pieces provide anchors for new growth [77].

Life cycles have also been proposed for anammox granules. Lu et al. [78] suggested that the largest anammox granules eventually break and provide material for forming new granules in a life-cycle type pattern. Across all granule types, there is a general consensus that small granules are considered ‘young’ and tend to break apart as they grow in size due to various factors such as shear force, internally trapped gas, and abrasion. It is then possible that granule pieces or single cells released from granules proceed to form the basis for new granules. This cycle of growth and breakage, along with the formation of new flocs and granules from individual cells and subsequent granule growth, could approximately explain the process of granulation across all three sludge types.

#### 2.1.5 Colloidal aggregation and the XDLVO theory

**Fig. 2:**
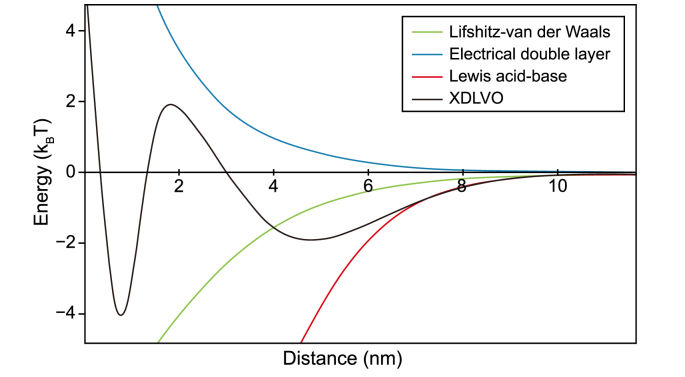
Example values for XDLVO interactions.

**Fig. 3:**
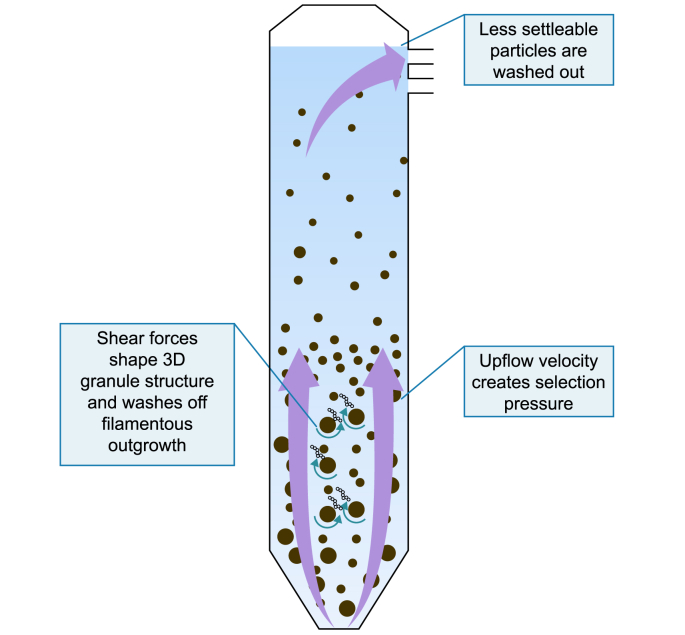
Selection pressure and shear force in an upflow reactor configuration.

Borrowing from the field of interface and colloidal science, researchers have attempted to explain granulation (and general bacterial adhesion) by simplifying the system to charged particles or surfaces interacting through a liquid medium. The current dominant thermodynamic model describing such interactions is the eXtended Derjaguin, Landau, Verwey, and Overbeek (XDLVO) theory [79]. According to the XDLVO theory, aggregation in water is mediated by the combination of Lifshitz-van der Waals forces, electrical double-layer interactions, and Lewis acid-base interactions (Fig. 2). As these act at different distances, their sum creates a characteristic interaction energy curve of which a conceptual example is shown in Fig. 3.

In principle, each of the XDLVO energy components can be calculated for two or more interacting particles or cells according to their surface characteristics and the qualities of the medium. However, only a few authors have successfully estimated the XDLVO interaction energy driving granulation from experimental measurements in anaerobic, aerobic, and anammox granular sludge. For example, Liu (2010) [80] used surface charge (*Zeta* potential and zero point of charge) and hydrophobicity (contact angle) to estimate the XDLVO interaction energy in both anaerobic and aerobic sludge. The authors could then mathematically explain how different EPS fractions influenced granulation according to their physicochemical properties.

Yuan et al. [81] used similar measurements in aerobic sludge, suggesting that the XDLVO interaction energy is mainly driven by Lewis acid-base interactions in the initial phase of granulation; and that the total XDLVO energy limits the formation of novel aggregates from forming during the sludge maturation phase.

Anammox granular sludge and its taxonomical composition have also been explained in accordance with the XDLVO theory by Ali et al. [82]. The authors found that the cell membrane of *Candidatus* Brocadia sinica, *Candidatus* Jettenia caeni, and *Candidatus* Brocadia sapporoensis, the three major anammox bacteria, have a negative (attractive) XDLVO interaction energy under their experimental conditions. Furthermore, they found that *Ca.* B. sinica has both a higher relative abundance in samples in granular sludge and a lower XDLVO interaction energy.

Fundamentally, the XDLVO interaction energy and its components overlap and are partially influenced by other factors discussed above, like the role of EPS and divalent cations. However, the XDLVO theory gives a comprehensive theoretical and mathematical framework for granulation. In future studies, the XDLVO theory and similar physicochemical theories of particle aggregation should be used more often, especially in light of recent advances in computational modelling. In particular, we suggest future research include measurable and controllable parameters of wastewater treatment processes, such as ionic strength or temperature in physicochemical aggregation models, allowing researchers and plant operators to explore how these parameters affect granulation.

### 2.2 Operational granulation theories

#### 2.2.1 Selection pressure

The concept of selection pressure with respect to granulation refers to the continual selection of dense, well-settling sludge and the washout of lighter sludge particles (Fig. 3) [83]. In continuous, methanogenic bioreactors, the selection pressure primarily depends on the upflow velocity (V_up_), determined by the influent flow and recycling rates. UASB bioreactors generally have V_up_ below 4 m h^−1^ and often operated below 1 m h^−1^ [84], whereas expanded granular sludge bed (EGSB) bioreactors are typically operated at V_up_ > 4 m h^−1^ [85]. High V_up_ (i.e., increasing selection pressure) causes dispersed, light sludge flocs and particles to be washed out, whilst heavier sludge is retained and subsequently grows, forming a well-settling sludge bed [62]. Increasing V_up_ increases selection pressure in the system and promotes granulation, often by stimulating EPS production [24,28].

The anammox process is also generally conducted in continuous systems, meaning that selection pressure is determined by upflow velocity. Increasing V_up_ and, thus, selection pressure has been shown to correlate with increased size and stability of anammox granules [85] and result in selective washout of smaller, less settleable biomass [86]. Since the growth rate of anammox biomass is low, these bioreactors are generally operated with less selection pressure under conditions of lower upflow, especially during start-up, to avoid excessive washout [15].

Since most aerobic granular sludge systems are operated as SBRs, settling time and aeration rate — rather than upflow velocity — determine selection pressure. Settling time is key to achieving fast aerobic granulation [87]. Liu and Tay [87] proposed a granulation mechanism whereby poorly settling flocs are washed out of bioreactors quickly due to high initial selection pressure. The remaining well-settling sludge was then less concentrated and was stressed by the resulting high OLR (8–12 g COD L^−1^ d^−1^) to remaining biomass ratio. This high load caused increased production of EPS, promoting granulation. The aeration rate also exerts a strong selection pressure on aerobic granulation, and increasing the aeration rate can improve granulation [88]. Settling time has also been determined as the main selection pressure in aerobic bubble column bioreactors [89].

While the physical effects of selection pressures, such as upflow velocities and settling times on granulation, are well established (i.e., selection of denser granules), the role of selection pressure in shaping the granule microbial community composition is unclear. It is often stated that high selection pressures selectively wash out organisms not adept at forming granules [90]. However, other studies have indicated that the microbial populations washed out of a reactor during granulation are simply proportional to the community inside the reactor [91]. Well-defined temporal studies at different degrees of selection pressures (e.g., V_up_) are required to determine the impacts of selection pressure on microbial community composition.

The causes (settling time or upflow velocity) and extent of selection pressure may vary with bioreactor and biomass type, and there are still some unknowns with regard to its role in microbial community selection. However, the underlying principles remain the same: higher selection pressure generally leads to better-settling biomass and improved granulation.

#### 2.2.2 Shear force

Like selection pressure, the shear force is essentially a function of bioreactor hydrodynamics, including liquid and gas upflow velocity (Fig. 3). Strong shear forces (8.28 s^−1^) have been shown to improve granulation in methanogenic bioreactors by stimulating the production of EPS rich in proteins [92]. However, in the same study, very strong or “violent” shear forces (12.42 s^−1^) were shown to negatively influence granulation. In addition, some research has indicated that in the right conditions, high shear forces are not always required for granulation. For example, granulation was achieved with low-strength wastewaters (340 mg COD L^−1^) under conditions of low shear forces [93]. Therefore, appropriate shear forces for granulation may vary depending on bioreactor conditions.

Increased shear force has been shown to cause an increase in the protein fraction of EPS in anammox granules and promote granulation [94]. Numerous studies have also reported that the EPS production rate in aerobic granules depends on shear force [95,96]. The influence of shear force on EPS production appears common among all granular sludge types [92,[95], [96], [97]]. Based on the above evidence, microorganisms respond to the stress of increasing shear force by producing EPS to aid aggregation and avoid washout from the bioreactor.

Liu and Tay [98] emphasized the importance of hydrodynamic shear force in shaping granular sludge (aerobic and methanogenic) and provided a comprehensive four-step mechanism for granulation. It was proposed that the final step in the granulation process involved steady growth, where hydrodynamic shear forces in the bioreactor shape the three-dimensional structure of the aggregate. Several other studies have reported the role of shear force in shaping newly formed granules into dense spherical aggregates by preventing the attachment of loose filamentous organisms on the granule surface [13,40]. Hence, shear force may have an important role in the initial stages of granule formation (stimulation of EPS production) and in forming granules into dense settleable entities.

### 2.3 Microbial granulation theories

#### 2.3.1 Key microbial species in granulation

The idea that microorganisms form a matrix in which other cells can embed is common to all granule types. This may be via filamentous growth morphologies and/or the production of an EPS matrix. There often appear to be one or several organisms that are of key importance for initiating granulation. One widely accepted aspect of methanogenic granulation is the importance of filamentous *Methanosaeta* sp., thought to provide a branched growth network within which other cells can embed [99]. Subsequent expansion of the aggregate occurs via microbial growth, and shear forces (caused by liquid and gas upflow) shape the aggregate into a dense sphere [19,20].

In contrast to methanogenic granules, no single organism has been universally found to dominate or be considered essential for aerobic granulation [3]. This may be because heterotrophic diversity is much higher than methanogenic diversity, or aerobic systems are more often operated for multiple processes, such as nitrogen, phosphorus and carbon removal [100]. Several EPS-producing bacteria have been suggested as important for aerobic granulation, including *Arcobacter*, *Aeromonas*, and *Flavobacterium* [101]; *Rhodanobacter* [100]; *Thauera* and *Zoogloea* [102], and *Agrobacterium* [103]*. Acinetobacter* sp. was also suggested in multiple studies to facilitate the initial aggregation of flocs into aerobic granules by acting as a “bridging” organism by linking flocs together with EPS [104,105]. *Candidatus* Accumulibacter has also been identified as important for aerobic granulation in multiple studies [106,107].

There are few temporal studies of anammox granular sludge communities during the initial stages of granulation. Many studies have identified the anammox bacteria, i.e., Planctomycetes, as the dominant organisms in anammox granules, with relative abundances as high as 80% [108]. The Planctomycetes generally form an internal core, suggesting that they may be involved in initiating granulation. However, Luo et al. [109] examined the microbial communities in size-resolved anammox granules and found that smaller granules (<1 mm) were dominated by ammonia oxidising bacteria (AOB), suggesting those bacteria are important in the early stages of granule formation, but become less important when granules grow. Anammox bacteria, AOB and nitrite-oxidising bacteria (NOB) are typically the dominant groups in anammox granular sludge; however, *Chloroflexi* is often found at relatively high abundances of 2–13% [[109], [110], [111]] and thought to have a role in maintaining granule structural integrity [112]. More research is required, particularly on actively growing anammox granules, to make stronger conclusions about which organisms initiate granulation. Microbial community data during the initial stages of anammox granulation from flocculent biomass or in size-resolved granules may help identify founder organisms.

Key to all microbiological-based granulation mechanisms is the presence of a key organism(s) at the outset of granule formation, often involved in producing a matrix of EPS or filamentous growth. While *Methanosaeta* sp. is widely accepted to initiate methanogenic granulation, we do not yet know a “founding” microorganism for anammox granules. Similarly, no single organism is accepted as universally responsible for granulation in aerobic systems, likely due to greater heterotrophic diversity. It is also apparent that EPS, rather than filamentous growth, is more important for initiating granulation in aerobic [100,101] and anammox [26,113] granules, whereas filamentous organisms are more important for methanogenic granulation [99].

#### 2.3.2 Layered structure

The concentric layering of biomass has been widely discussed in many in-depth granulation studies. A layered structure for methanogenic granules was first suggested by MacLeod et al. [114] and later by Ahn [115], in which aggregations of *Methanosaeta* sp. formed a central core in a layered sphere. Other functional groups, such as fermentative, acidogenic and acetogenic bacteria, form concentric layers around the archaeal core of methanogens [19,116]. Several studies later confirmed using 16S rRNA-targeted fluorescence *in situ* hybridization (FISH) [117].

Layers have also been observed in aerobic granules, and it is generally accepted that they contain aerobic outer layers, surrounding an anoxic middle layer with an anaerobic, or inert, core [[118], [119], [120]]. Lv et al. [121] used denaturing gradient gel electrophoresis DGGE fingerprinting and high-throughput amplicon sequencing to study thin sections of aerobic granules. This revealed a core dominated by anaerobic organisms, such as the *Rhodocyclaceae*, and an outer layer of aerobic and facultative anaerobic organisms, such as *Microbacteriaceae*, *Sphingobacteriaceae*, and *Moraxellaceae.* The spatial distribution of microorganisms in aerobic granules can be more complex than methanogenic granules since these are typically applied for nitrogen and/or phosphorus removal and chemical oxygen demand (COD) removal. The NOB and AOB are often located in the outer layer of aerobic granules, where oxygen is more readily available. Meanwhile, denitrifying bacteria are located in the anoxic layers [3,122].

Vlaeminck et al. [123] formed a comprehensive anammox granulation model based on the spatial organization of key anammox groups. It was hypothesized that aggregation of single cells leads to the formation of flocs, which grow and develop an outer rim of aerobic ammonium oxidisers resulting in an anoxic core of anammox bacteria [123]. While there does often appear to be a layered structure in anammox granules, the anammox bacteria at the centre of the granule tend to be organised according to a “cell-cluster” type internal architecture, which appears to be a result of the aggregation of several smaller anammox granules into a larger aggregate, followed by subsequent concentric growth [33,110,124].

The observation of similar concentric layer structures across all granule types implies that a similar growth mechanism may occur. Concentric growth suggests the formation of an initial nucleus is followed by either outward growth from the centre or attachment of cells at the granule surface. In either way, the increasing granule size leads to redox gradients from the granule core to the surface, e.g., gradients in oxygen concentration. These gradients create different environmental niches, allowing various organisms to proliferate at different granule depths.

#### 2.3.3 Granule size and granule growth

Granule size is a frequently measured characteristic often used to assess the progress or stage of granulation. Granules typically range between 0.5 and 4 mm [3]. Granule growth is often associated with improved performance in aerobic [125], methanogenic [126], and anammox granules [127]. However, there does appear to be a limit at which increasing granule size becomes detrimental. This can lead to dead zones at the centre of the granule, where cells either die off or become inactive due to substrate limitation [128], therefore taking up space which could have been occupied by active microorganisms.

**Fig. 4:**
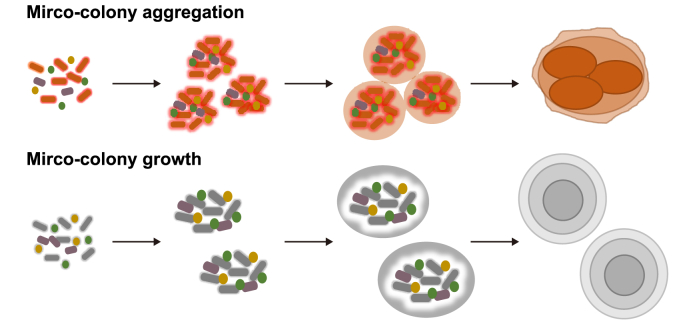
Mechanisms of granule development according to the micro-colony growth and aggregation theories.

In general, there are two types of granulation mechanisms based on granule size: (1) micro-colony aggregation and (2) micro-colony growth (Fig. 4). Micro-colony aggregation involves the initial formation of small cell clusters, often termed flocs, micro-colonies or micro-granules, which aggregate and form a larger granule that subsequently expands with further cell attachment and growth [33,50,124]. This results in internal cell clusters [78] within larger granules, each of which was originally separate micro-colonies [50]. Micro-colony growth also involves the initial formation of small cell clusters but is followed by the continual growth of each such cluster, without further aggregation, and generally leads to the expansion of a concentrically organised [117,121] microbial community.

Micro-colony aggregation is more commonly reported in anammox granules and leads to mature granules with internal sub-units [33,129]. Despite the abundance of micro-colony aggregation-based theories in anammox granulation, there is some evidence of micro-colony growth, resulting in concentric architectures [123]. Granule size is thought to play an important role in determining the level of anammox activity, with larger granules being more active and containing a higher relative abundance of Planctomycetes [130,131]. It has been proposed that, as granules grow, more strict anaerobic conditions develop at the core of the granule, leading to the proliferation of anammox bacteria [132]. Metatranscriptomic evidence has also supported this hypothesis, as the expression of key anammox metabolic genes and the production of transport proteins increased with granule size [133].

Granulation mechanisms proposed for methanogenic granules tend to be based more on micro-colony growth than aggregation of separate micro-colonies, resulting in a layered morphology [114,115]. Granule size can greatly impact microbial community composition in methanogenic granules. For example, microbial communities in methanogenic granules have been shown to shift from an acidogen dominated communities in small granules or flocculent sludge [75,134] to more typical methanogenic communities in larger granules [135,136]. Trego et al. [137] even concluded that granule size had a greater impact on the active microbial community of methanogenic granules than the availability of specific methanogenic substrates.

Zhou et al. [138] proposed a micro-colony aggregation mechanism in aerobic granules, whereby small “bioflocs” aggregated to form individual small granules, which then grew successively larger. However, micro-colony growth mechanisms resulting in concentric architecture have been proposed more frequently in aerobic granulation [120,121]. Granulation by both micro-colony aggregation and micro-colony growth was observed to occur side by side in an aerobic granular sludge system used for biological phosphorus removal [139], indicating both contribute to granule formation.

## 3 Different features among granule types

**Fig. 5:**
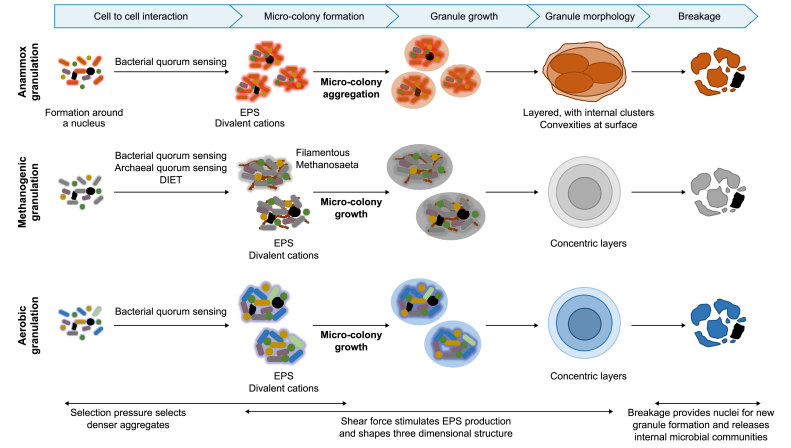
Graphical representation of similarities and differences between granulation mechanisms of the reviewed granule type.

While all three sludge types share multiple characteristics (Fig. 5), it must be acknowledged that some differences in granules and possible granulation mechanisms may exist, and a singular unified theory of granulation may not be possible. Some examples include the distinct anammox granule morphology, the role of DIET in methanogenic granules, and different quorum sensing systems. These differences should be taken into account when working with individual sludge types, as they may allow more targeted improvement of granulation and bioreactor operation in addition to the general concepts.

### 3.1 Anammox granule morphology

In contrast to aerobic and methanogenic granules, anammox granules typically have an internal structure comprising separate cell clusters [33]. The outer surface of anammox granules is often rougher than other granule types, with bumps and convex protrusions, as quantified by Kang et al. [129]. A ‘budding’ mechanism, which may account for this structural difference, was proposed by Ref. [123], whereby the growth of anaerobic ammonium oxidisers at the core of the granule causes protrusions at the outer surface. However, this rough surface morphology may also result from a micro-colony aggregation mechanism, as discussed in Section 2.3.3. Regardless of the causes, these morphological differences in anammox granules indicate that the formation mechanism is slightly different than in aerobic or anammox granules.

### 3.2 DIET in methanogenic granules

Syntrophic interactions between microorganisms in AD are often facilitated by reduced intermediate molecules, such as hydrogen or formate [140]. For example, syntrophic propionate-oxidising organisms produce molecular hydrogen, which is used by hydrogenotrophic methanogens [141]. In such partnerships, the methanogen is essential in maintaining low H_2_ partial pressures, allowing volatile fatty acids (VFA) oxidation to proceed as thermodynamically feasible reactions [142].

Electrons can also be transferred directly from one microorganism to another, by DIET, without exogenous electron carriers [143]. This was observed initially in co-cultures of *Geobacter* sp. and *Methanosaeta* sp. [144]. Close physical association between DIET partners is required to transfer electrons through membrane-bound cytochromes or conductive pili [145], and microorganisms engaging in DIET have been shown to favour aggregated growth [144]. The process of DIET is now considered to be common in anaerobic granules [143]. However, it is still unclear whether DIET enhances granulation or whether granulation enhances DIET.

Shrestha et al. [146] found no detectable conductivity in aerobic nor anammox granules, suggesting the absence of DIET in such biofilms. However, DIET has been speculated in anammox-sulphur systems, where anammox bacteria oxidise ammonium and directly transfer electrons to sulfate-reducing bacteria [147]. Extracellular electron transfer (EET) through conductive materials has been conclusively demonstrated in anammox granules. For example, recent studies have identified EET mediated by pyrite in anammox granular sludge [148], and the ability of diverse anammox bacteria to use carbon-based insoluble extracellular electron acceptors for the oxidation of NH_4_^+^ [149] has also been demonstrated. These studies suggested that anammox bacteria can perform EET, but direct evidence for DIET is absent in unamended granules. A range of conductive additives have also been assessed in promoting EET in methanogenic bioreactors [150]. Various additives, such as granular activated carbon, graphite and biochar, along with other carbon-based materials, were shown to enhance bioreactor performance [151,152], and may have an effect on the granulation mechanism.

There is no current evidence to suggest that true cell-to-cell DIET takes place in aerobic or anammox granules, but the role of DIET in methanogenic granulation should be taken into account in any granulation theory. Also, the role of conductive additives must be considered in both methanogenic and anammox granulation.

### 3.3 Unique quorum sensing systems

The role of quorum sensing (QS) differs among sludge types. In aerobic granulation, increases in acyl homoserine lactone (AHL) signal molecules positively correlate with increased EPS production and improved granulation [153]. Regulation of QS in aerobic systems has been attempted through addition of exogenous AHL signal molecules [153], bioaugmentation with AHL-producing strains [154], and even the addition of AHL-containing supernatant from mixed cultures [155]. However, some knowledge gaps prevail, such as the role of quorum quenching in granule disintegration, as highlighted by Sarma et al. [120] in a review on aerobic granulation.

Anammox granules also demonstrate quorum sensing [156], and AHL concentrations positively correlated with improved anammox activity [157] and improved granule settleability [155]. Inactivation of AHLs and interference with AHL receptors in anammox granular sludge reduced anammox activity and influenced protein:polysaccharide ratios in EPS, resulting in granule disintegration [158]. This indicates that QS is essential for maintaining stable anammox granules and has a similar role to aerobic granulation.

In methanogenic granulation, the role of archaea in QS is still largely unknown [159]. A methanogen-specific QS system with modified AHLs has been identified in *Methanosaeta harundinacea* 6Ac [160]. Production of these AHLs causes a shift from growth as short cells to filamentous cells, which may benefit granulation [160]. Bioaugmentation of this strain, along with its specific AHLs, has been shown to improve granulation and reduce sludge washout [161]. Therefore, QS in methanogens may induce filamentous morphologies and initiate methanogenic granulation. This indicates that quorum sensing has a slightly different role in aerobic and anammox granulation than methanogenic granulation. Methanogenic QS may provide a cellular matrix for granule formation, whereas bacterial QS in aerobic and anammox granules may provide an EPS matrix rich in proteins. Both bacterial and archaeal QS occur in methanogenic granulation, but the relative importance of each is unclear. Therefore, quantifying the effect of bacterial versus methanogenic QS in methanogenic granulation requires further investigation.

## 4 An ecological approach to studying granulation

Since a single, universally present, *Methanosaeta*-like equivalent is unlikely to occur in aerobic granulation, and the modes of growth (i.e., micro-colony aggregation or growth, see Fig. 4) can vary between granule types, thus developing a universal theory of granulation is, at present, not possible. However, one aspect of granulation, which is understudied and may contribute to understanding the inherent mechanisms, is microbial ecology. Rather than simply identifying and listing specific organisms present in granular biomass, a broader approach should be applied to examine ecological theories or phenomena that may influence granulation. This would be particularly useful in actively forming granules rather than already established granules and would address an important research gap. Concepts, such as interspecies interactions, community assembly or succession, could all be used in this context since they can directly affect microbial aggregation. This may provide additional commonalities between sludge types and contribute to our understanding of granulation across all three sludge types considered here.

### 4.1 Interspecies interactions

Granules contain redox gradients (e.g., of oxygen) [119], and ecological niche spaces, which are required to accommodate all of the microorganisms necessary for the degradation of a wide range of compounds. However, it is currently unclear whether granulation facilitates increased interspecies interaction or interspecies interaction facilitates granulation. It is possible that both statements are true and interspecies interactions, such as commensalism, competition, and mutualism, play a role in each scenario. For example, the mutualistic relationship between syntrophic VFA-oxidising bacteria and hydrogenotrophic methanogens [142] may promote close physical associations, thus facilitating granulation. However, it may also be the case that granulation occurs independently of these interactions and these associations a promoted after granules have established. The role and extent of such interactions require investigation in actively forming and growing granules to determine if they influence granulation.

Antagonistic interactions, such as competition, also occur in granular communities, for example, between methanogens and sulfate-reducing bacteria (SRBs) [162]. While much of our understanding of these interspecies interactions stems from co-culture studies, a more effective approach for investigating such interactions in mixed cultures is network analysis. This analytical method is commonly employed to uncover interspecies interactions in diverse microbial communities [163]. Such analysis, applied specifically in the context of granulation, could unveil patterns of interspecies interactions that are common across granule types and aid in developing a universal granulation model. However, it is important to note that the interactions identified using these methods are only mathematical in nature and do not take into account any biological significance. Therefore, complementary techniques such as transcriptomics, metabolomics or stable isotope probing may be required to conclusively identify interspecies interactions.

Some ecological interactions, such as predation, are underexplored in granular biomass, particularly in relation to granulation. Predation by *Bdellovibrio bacteriovorus* can influence traditional biofilm morphology, by promoting denser cell packing at the centre of the biofilm [164]. In contrast, predation by *B. bacteriovorus* at the periphery of biofilms results in looser cell structures, making the biofilm more susceptible to immigration [164]. Although these findings were from surface-attached, single-species biofilms, similar interactions may occur in granules. For example, the outer layers of granules, or newly formed granules, are often less densely packed and may be disproportionately affected by predation, allowing for the immigration of other taxa and diversification of the granule community. Predation by protozoa in a methanogenic UASB increased methanogenic activity and prokaryotic diversity, although granulation was not studied in detail [165]. Predation by protozoa or parasitism by bacteriophages have previously been shown to correlate with improved aerobic granulation, and the absence of protozoa delayed the onset of granulation [166]. While some useful findings have been made on predation in granular systems, there is still huge research potential in this area, especially in relation to its role in shaping early granule communities.

### 4.2 Community assembly and community succession

Since specific interspecies interactions are difficult to study in complex microbial communities, broader measurements, such as community assembly and community succession, could be assessed to provide an ecological perspective on granulation. Community succession of functional groups in growing methanogenic granules has previously been described [135], and functional gene succession in anammox granular sludge indicated that EPS-producing genes were associated with improved aggregation [167]. A similar approach examining the succession of functional groups or genes in more studies across all granule types could provide a consensus on granule formation mechanisms.

Community assembly is often studied using null modelling approaches to assess the relative contributions of stochastic or deterministic forces in microbial community formation. These approaches have been applied to surface-attached biofilms, including stream biofilms [168] and micro-plastic-associated biofilms [169]. For example, it was demonstrated that microplastic-associated microbial communities in marine environments assemble stochastically through random collisions with microorganisms rather than through deterministic processes related to the environment or microplastic characteristics [138]. Ezzat et al. [168] found that benthic stream biofilms were more heavily influenced by deterministic processes than streamwater biofilms. The authors attributed this to increased residence time for cells in benthic biofilms, facilitated by an EPS matrix, which allowed the construction of different niches and the domination of niche-related deterministic processes [168]. Similar approaches, along with fluorescent *in situ* hybridization FISH microscopy, were applied to study surface-attached anammox biofilms grown on flat carrier materials [170]. This led to the development of a three-stage model for biofilm growth, where growth stages were defined by stochastic and deterministic processes [170].

The approaches outlined above could be applied to granular biomass of all types and would be appropriate for the development of a universal granulation model. To date, a combination of deterministic and stochastic community assembly processes has been observed in methanogenic granular biomass [171,172], with their relative contributions assessed in aerobic and methanogenic granulation [136]. Reproducible patterns of community assembly have even been demonstrated in replicate bioreactors [104]. Currently, deterministic processes appear to play a more significant role than stochastic processes in community assembly of granules, but a degree of stochasticity is always present. Additional studies across all granule types and in various bioreactor configurations, especially in actively growing granules, are required to reliably predict patterns of community assembly during granulation.

### 4.3 A single granule approach

To date, much of the sequencing data from granular biomass has been obtained by extracting DNA from multiple granules. However, a single-granule approach may be more appropriate for studying granulation. Existing studies of single granules have shown considerable variation between individual granule communities [173,174]. However, neither Kuroda et al. [174] nor Leventhal et al. [173] delved into potential granulation mechanisms. Working at the single single-granule level would allow more defined data to be obtained at different stages of granulation. For example, rather than obtaining the mean values for various microbiome metrics across multiple granules, more high-resolution data for individual granules could be obtained. This more defined data would be appropriate for developing a granulation model similar to the stepwise process, widely recognised for traditional, surface-attached biofilms [175]. Such a model may allow optimal life stages of granules to be identified and associated with bioreactor performance. In traditional biofilms, five main steps have been outlined: initial attachment, EPS production, early biofilm formation, maturation, and dispersal [175]. There are similarities here with a lot of the previously discussed granulation mechanisms. For example, the establishment of EPS matrices and maturation during three-dimensional growth. Studying single granules at different stages of growth and maturation would remove a lot of the noise, which may originate from multiple granules and be more suitable for developing a stepwise, defined granulation model. This could be used to steer operational parameters, such as upflow velocity or OLR, to regulate granule growth. For example, by identifying and maintaining the stage of granule growth characterized by maximum diversity and functional redundancy, bioreactors can become more resilient to operational shocks.

## 5 Conclusions

Several unifying concepts have been identified across the three examined granule types. Selection pressure, shear forces, EPS (particularly protein content), divalent cations, and inorganic nuclei play similar roles in each sludge type. These concepts appear important during the early stages of granulation, leading to the initial formation of micro-colonies. However, some differences do exist in the early stages of granulation. For example, the role of filamentous organisms, such as *Methanosaeta* sp., in methanogenic granulation is not replicated in other granule types. Instead, anammox and aerobic granules rely more on an EPS matrix. In addition, the diverse microbiomes in aerobic granular sludge make it unlikely that a single organism, such as *Methanosaeta* sp., universally drives granulation. A major difference is observed in growth modes. Whereas initial micro-colonies grow independently, forming methanogenic and aerobic granules with concentric internal layering, anammox micro-colonies tend to aggregate together, resulting in a slightly different internal structure. Given these differences, developing a universal granulation mechanism may be impossible. Instead, accepting and acknowledging these differences is important when interpreting data from disparate sludge types. One area which requires more investigation in all sludge types is microbial ecology. Focusing on broader patterns and interactions in microbial communities, rather than simply identifying which organisms are present, may offer valuable insights into granule formation. In addition, adopting a single-granule approach could help identify distinct growth stages, facilitating the implementation of optimal operational strategies.

## Credit author contribution statement

**Simon Mills**: Conceptualization, Writing - Original Draft, Visualization. **Anna Christine Trego**: Conceptualization, Writing - Review & Editing. **Marco Prevedello**: Writing - Original Draft, Visualization. **Jo De Vrieze**: Writing - Review & Editing. **Vincent O’Flaherty**: Writing - Review & Editing. **Piet Lens**: Writing - Review & Editing, Supervision. **Gavin Collins**: Conceptualization, Writing - Review & Editing, Supervision.

## Declaration of competing interest

The authors declare that they have no known competing financial interests or personal relationships that could have appeared to influence the work reported in this paper.
